# Nutritional services in primary healthcare in the Republic of Srpska, Bosnia and Herzegovina: strengthening prevention through multidisciplinary approaches

**DOI:** 10.3389/ijph.2026.1609839

**Published:** 2026-10-02

**Authors:** Jelena Niškanović, Dragana Stojisavljević, Dijana Manigoda

**Affiliations:** 1 Faculty of Philosophy, University of Banja Luka, Banja Luka, Bosnia and Herzegovina; 2 Public Health Institute of the Republic of Srpska, Banja Luka, Bosnia and Herzegovina; 3 Faculty of Medicine, University of Banja Luka, Banja Luka, Bosnia and Herzegovina

**Keywords:** barriers, nutrition services, preventive care, primary healthcare, public health

## Abstract

**Objectives:**

This study aimed to assess the provision of nutritional services in primary healthcare facilities (PHCs) in the Republic of Srpska, Bosnia and Herzegovina, with a focus on differences between departments, namely Family Medicine, Pediatrics, and Gynecology.

**Methods:**

A mixed-methods design was applied, combining a cross-sectional quantitative survey and qualitative focus group discussions. The quantitative component included 50 PHC facilities, while qualitative data were collected through three focus groups with healthcare professionals.

**Results:**

Services related to the improvement of nutrition and the prevention of non-communicable diseases (NCDs) were provided by 92.4% of PHCs, while 80.5% monitored the nutritional status of the population. Key barriers included lack of time, insufficient staffing, limited financial incentives, and the absence of standardized guidelines. Significant differences in the reported lack of time were found between departments (F = 4.402, p < 0.05), specifically between the Gynecology and Family Medicine departments.

**Conclusion:**

Findings from healthcare personnel highlight the need for a systemic reorganization of service provision, the development of standardized nutritional guidelines, and the strengthening of preventive services through multidisciplinary approaches.

## Introduction

The UNICEF Nutrition Strategy 2020–2030 envisions “a world where all children, adolescents and women realize their right to nutrition”, grounded in the Convention on the Rights of the Child, which recognizes adequate nutrition as a fundamental human right. The strategy aims to promote diets, services, and practices that support optimal growth and development while contributing to the achievement of the Sustainable Development Goals, particularly those related to ending all forms of malnutrition [1].

Poor dietary habits represent a major global health risk. It is estimated that approximately one in five deaths worldwide is attributable to suboptimal diet, accounting for approximately 11 million deaths annually [2]. At the same time, the increasing prevalence of overweight and obesity has become a significant public health challenge, contributing to the global burden of non-communicable diseases (NCDs) and causing approximately four million deaths annually [3, 4].

Malnutrition remains a persistent and multifaceted issue, particularly among children. Nearly two-thirds of children aged 6–23 months do not receive adequate nutrition, leading to both short- and long-term health consequences [5]. Malnutrition manifests in several forms, including stunting, micronutrient deficiencies, and the growing prevalence of childhood overweight and obesity, all of which have significant implications for health across the life course [6–8].

In recent years, global initiatives such as the Scaling Up Nutrition Movement, the World Health Assembly nutrition targets, and the United Nations Decade of Action on Nutrition have placed nutrition at the center of development agendas, emphasizing the need for stronger health system responses [7]. Primary healthcare (PHC) systems play a key role in delivering nutrition-related services due to their accessibility and focus on prevention. However, despite the recognized importance of nutrition in PHC, services such as nutritional assessment, monitoring, and counseling remain insufficiently integrated and underfunded in many settings [9, 10].

Several barriers to effective service provision have been identified, including limited training of healthcare professionals, lack of standardized guidelines, insufficient financial incentives, and inadequate health information systems [11–14]. Strengthening workforce capacity, promoting multidisciplinary approaches, and improving organizational frameworks are therefore essential for enhancing nutrition services within PHC [15–18]. Additionally, improved monitoring systems and the use of digital tools may support the better planning and delivery of nutrition-related interventions [19].

Nutritional services constitute a fundamental component of PHC, particularly in the prevention and management of NCDs, offering a unique opportunity for the early detection of nutritional risk factors, continuous patient engagement, and the implementation of preventive interventions at the population level [20–22]. In the Republic of Srpska, Bosnia and Herzegovina, evidence on the provision of nutritional services within primary healthcare remains limited, particularly across departments such as Family Medicine, Pediatrics, and Gynecology [23].

Therefore, the aim of this study was to assess the provision of nutritional services in primary healthcare facilities, with a particular focus on practices in the Family Medicine, Pediatrics, and Gynecology departments. This study also aimed to identify key barriers, and explore potential solutions for improving service delivery at the PHC level.

## Methods

### Study design

The present study used a mixed-methods design, combining quantitative and qualitative research methods. The quantitative component was organized as a cross-sectional study among PHCs, while the qualitative component was carried out through focus groups with medical personnel (medical doctors and nurses). The two components were conducted concurrently in December 2023, and the findings were integrated during the interpretation stage.

### Sample and participants

The sample for the quantitative research included all public healthcare centers (54 primary health centers and the outpatient health unit East Stari Grad) in the Republic of Srpska, Bosnia and Herzegovina, that provide services at the primary healthcare level in accordance with Article 36 of the Law on Healthcare of the Republic of Srpska [23]. More precisely, the target population consisted of lead physicians from the Family Medicine, Gynecology, and Pediatric departments of these facilities. All of the 54 healthcare centers and the outpatient health unit have Family Medicine departments employing 619 medical doctors, while 44 facilities have Pediatric departments with 82 medical doctors employed, and 36 facilities have Gynecology departments with 62 medical doctors employed [24]. By the end of data collection, 50 healthcare facilities had submitted complete questionnaires. The detailed sample structure and response rate are presented in Table 1.

**TABLE 1:** Survey sample and response rate by target group (Republic of Srpska, Bosnia and Herzegovina, 2023).

Target population/PHC department	Number of facilities included in the sample	Number of completed questionnaires	Response rate (%)
Family medicine	55	49	89.1%
Pediatrics	44	37	84.1%
Gynecology	36	32	88.9%

The qualitative part of the study was carried out through focus groups involving representatives from the selected primary healthcare centers, that is, representatives of the Family Medicine, Pediatric, and Gynecology departments. The primary healthcare centers were selected according to the size of the local community and the geographical distribution of the facilities. After initial consideration, 12 facilities were invited to participate in the qualitative study and to nominate one medical doctor and one nurse from each department to participate in the focus groups. Three focus groups were conducted: one with Family Medicine personnel, one with Pediatric personnel, and one with Gynecology personnel. The number of healthcare centers and the number of participants in each focus group are presented in Table 2.

**TABLE 2:** Focus groups structure according to the target population of this study (Republic of Srpska, Bosnia and Herzegovina, 2023).

Focus group with representatives of	Number of primary healthcare centers participating in the study	Number of participants at each focus group
Family medicine	12	24
Gynecology	7	13
Pediatrics	7	10

### Instruments

The survey questionnaire consisted of 12 questions and one scale encompassing data related to nutritional practices, such as 1) the provision of an assessment of nutritional status (0-no/1-yes) and its related services, such as monitoring of anthropometric parameters (0-no/1-yes), use of dietetic questionnaires (0-no/1-yes), assessment of laboratory parameters (0-no/1-yes), and assessment of clinical parameters (0-no/1-yes); and 2) the provision of services related to the improvement of nutrition and the prevention of NCDs (0-no/1-yes) and its related services, such as nutritional counseling (0-no/1-yes), counseling on physical activity (0-no/1-yes), counseling for women of reproductive age (0-no/1-yes), breastfeeding counseling (0-no/1-yes), counseling for overweight and obese patients (0-no/1-yes), and early detection of overweight and obesity (0-no/1-yes). The dominant barriers to the provision of nutritional services were assessed on a 5-point Likert scale (1- not important at all, to 5- the most important) comprising six items: insufficient time, financial compensation, patient cooperation, patients’ knowledge of nutrition, identification of patients in need of nutritional counseling, and medical personnel’s skills related to nutritional counseling. The questionnaire was developed based on available literature and existing recommendations from similar research in the field of nutrition [9, 11, 12, 15, 16], and was pre-tested through a panel discussion with experts from PHCs in order to establish content validity and terminological clarity prior to full-scale data collection. The same version of the questionnaire was used across all PHC departments.

The focus group guide consisted of 11 questions covering three main topics: 1) assessment of the current scope and quality of nutritional services at the PHC level; 2) proposals for improving the quality of nutritional services at the PHC level; and 3) assessment of current intersectoral cooperation, together with concrete proposals for its improvement. The questions were designed to explore barriers and obstacles related to the provision of nutritional services and to gain insight into the possibilities and solutions for improving these services and preventing NCDs at the healthcare facility and local community levels.

### Data collection

The first phase of this study included a meeting with the directors of all primary healthcare centers in the Republic of Srpska, BiH, which was held on 7 December 2023, to present the purpose, objectives, and methodology of the research.

The second phase involved sending the questionnaires by post to the management of the primary healthcare centers and to the Family Medicine, Pediatric, and Gynecology departments in 55 primary healthcare facilities in the Republic of Srpska, BiH. Data collection was carried out between 8 and 27 December 2023, with continuous communication via email and telephone with all facilities that did not submit the questionnaires on time. Once the questionnaire had been completed in each department in a given facility, these were returned by post to the Public Health Institute of the Republic of Srpska.

The third phase involved organizing and implementing focus groups, which were held online via the Zoom platform in December 2023, concurrently with the collection of the distributed questionnaires. The qualitative research was preceded by the delivery of an informed consent form for participation in the research, along with key information about the aims and purpose of the research and Zoom access links, to the Family Medicine, Gynecology, and Pediatric teams in the primary healthcare centers selected to participate in the focus groups.

### Data analysis

The provision of nutritional services (monitoring of nutritional status and nutritional counseling) was analyzed using descriptive statistics, together with the chi-square test to assess differences between the departments of the PHCs. Analysis of variance (ANOVA) was used to compare the level of reported barriers to providing nutritional services across departments. Statistical significance was set at *p* < 0.05.

Thematic analysis was undertaken in the following phases: (1) data immersion, (2) data familiarization, (3) code generation, (4) selection of themes, (5) thematic review, and (6) final selection of themes. Qualitative data analysis was not based on any specific theory; rather, data were analyzed in order to identify the themes that emerged and thus to understand the barriers to, and the potential solutions, for the improvement of nutritional services.

### Ethical considerations

The Ministry of Health and Social Welfare of the Government of the Republic of Srpska (approval no. 11/08-505-193/23) and the Ethics Committee of the Public Health Institute of the Republic of Srpska (approval no. 501-1-16/23) granted consent for carrying out this research. All procedures performed in studies involving human participants were in accordance with the 1964 Declaration of Helsinki and its later amendments. Informed consent was obtained from all individual participants included in the study.

## Results

### Quantitative data analysis

A high percentage of PHC facilities (80.5%) monitored the nutritional status of the population, although this varied across departments. The Family Medicine (91.8%) and Pediatric (91.9%) departments demonstrated a significantly higher level of engagement in nutritional status monitoring than the Gynecology departments (50.0%) (χ^2^ = 26.043, df = 2, p < 0.001).

An analysis of the individual services related to the monitoring of nutritional status showed that PHCs most frequently monitored anthropometric parameters (75.4%), while approximately half of them (46.6%) assessed laboratory parameters related to nutritional status. A smaller percentage of facilities used dietetic questionnaires (28.0%) or assessed clinical parameters (20.3%). When these services were compared across departments, the Family Medicine and Pediatric departments demonstrated a significantly higher level of engagement in monitoring all parameters, with the exception of clinical parameters, than the Gynecology departments (p < 0.001) (Table 3).

**TABLE 3:** Monitoring of nutritional status of the population and related services across departments in primary healthcare facilities (Republic of Srpska, Bosnia and Herzegovina, 2023).

Variables	Pediatrics (n = 37)	Gynecology (n = 32)	Family medicine (n = 49)	Total (n = 118)	p
Monitoring of nutritional status of the population					​
No	3 (8.1%)	16 (50.0%)	4 (8.2%)	23 (19.5%)	<0.001
Yes	**34 (91.9%)**	16 (50.0%)	**45 (91.8%)**	95 (80.5%)	​
Monitoring of anthropometric parameters				​	​
No	4 (10.8%)	18 (56.3%)	7 (14.3%)	29 (24.6%)	<0.001
Yes	**33 (89.2%)**	14 (43.8%)	**42 (85.7%)**	89 (75.4%)	​
Conducting dietetic questionnaires					​
No	17 (45.9%)	**30 (93.8%)**	38 (77.6%)	85 (72.0%)	<0.001
Yes	20 (54.1%)	2 (6.3%)	11 (22.4%)	33 (28.0%)	​
Provision of laboratory parameters					​
No	17 (45.9%)	25 (78.1%)	21 (42.9%)	63 (53.4%)	0.004
Yes	**20 (54.1%)**	7 (21.9%)	**28 (57.1%)**	55 (46.6%)	​
Clinical parameters					​
No	27 (73.0%)	29 (90.6%)	38 (77.6%)	94 (79.7%)	0.171
Yes	10 (27.0%)	3 (9.4%)	11 (22.4%)	24 (20.3%)	​

Bold values indicate the department with the significantly higher proportion (p < 0.05).

Services related to the improvement of nutrition and the prevention of NCDs were provided by 92.4% of PHCs. Nutritional counseling was significantly more frequently provided in the Family Medicine (87.8%) and Pediatric (86.5%) departments than in the Gynecology department (50.0%) (χ^2^ = 18.318, df = 2, p < 0.001). A similar trend was observed for counseling on physical activity (χ^2^ = 9.928, df = 2, p < 0.01). Counseling for women of reproductive age was significantly more frequent in the Gynecology department (71.9%) than in the Family Medicine (30.6%) and Pediatric (21.6%) departments (χ^2^ = 20.687, df = 2, p < 0.001). Breastfeeding counseling and the early detection of overweight and obesity were significantly more frequent in the Pediatric department than in the other two departments (χ^2^ = 56.112, df = 2, p < 0.001; χ^2^ = 20.112, df = 2, p < 0.001), whereas counseling for overweight and obese patients was significantly more frequent in the Family Medicine departments (χ^2^ = 12.435, df = 2, p < 0.01).

Overall, among the services related to the improvement of nutrition and the prevention of NCDs, nutritional counseling (77.1%), counseling on physical activity (73.7%), and counseling for overweight and obese patients (66.1%) were more frequently provided by PHCs than the early detection of overweight and obesity (51.7%), breastfeeding counseling (46.6%), and counseling for women of reproductive age (39.0%) (Table 4).

**TABLE 4:** Provision of services related to the improvement of nutrition and the prevention of non-communicable diseases and related counseling across departments in primary healthcare facilities (Republic of Srpska, Bosnia and Herzegovina, 2023).

Variables	Pediatrics (n = 37)	Gynecology (n = 32)	Family medicine (n = 49)	Total (n = 118)	p
Provision of services related to the improvement of nutrition and the prevention of NCDs					​
No	1 (2.7%)	5 (15.6%)	3 (6.1%)	9 (7.6%)	0.114
Yes	36 (97.3%)	27 (84.4%)	46 (93.9%)	109 (92.4%)	​
Provision of nutritional counseling					​
No	5 (13.5%)	16 (50.0%)	6 (12.2%)	27 (22.9%)	<0.001
Yes	**32 (86.5%)**	16 (50.0%)	**43 (87.8%)**	91 (77.1%)	​
Provision of counseling on physical activity					​
No	8 (21.6%)	15 (46.9%)	8 (16.3%)	31 (26.3%)	0.007
Yes	**29 (78.4%)**	17 (53.1%)	**41 (83.7%)**	87 (73.7%)	​
Provision of counseling for women of reproductive age					​
No	29 (78.4%)	9 (28.1%)	34 (69.4%)	72 (61.0%)	<0.001
Yes	8 (21.6%)	**23 (71.9%)**	15 (30.6%)	46 (39.0%)	​
Provision of breastfeeding counseling					​
No	1 (2.7%)	23 (71.9%)	39 (79.6%)	63 (53.4%)	<0.001
Yes	**36 (97.3%)**	9 (28.1%)	10 (20.4%)	55 (46.6%)	​
Provision of counseling for overweight and obese patients					​
No	13 (35.1%)	18 (56.3%)	9 (18.4%)	40 (33.9%)	0.002
Yes	24 (64.9%)	14 (43.8%)	**40 (81.6%)**	78 (66.1%)	​
Provision of early detection of overweight and obesity					​
No	11 (29.7%)	26 (81.3%)	20 (40.8%)	57 (48.3%)	<0.001
Yes	**26 (70.3%)**	6 (18.8%)	**29 (59.2%)**	61 (51.7%)	​

Bold values indicate the department with the significantly higher proportion (p < 0.05).

Barriers to the provision of nutritional services were assessed on a five-point Likert scale (1 – not important at all to 5 – the most important). The most significant barriers were the lack of financial compensation for the provision of nutritional services by the Health Insurance Fund of the Republic of Srpska (Mean = 4.53, SD = 0.86), lack of time for counseling (Mean = 4.19, SD = 1.00), and patients’ knowledge of nutrition (Mean = 3.92, SD = 0.86).

ANOVA revealed significant differences between departments in the reported lack of time (F = 4.402, p < 0.05). A *post hoc* LSD test showed significant differences in mean values between the Gynecology (Mean = 3.68, SD = 1.21) and Family Medicine (Mean = 4.47, SD = 0.97) departments (Figure 1).

**FIGURE 1:**
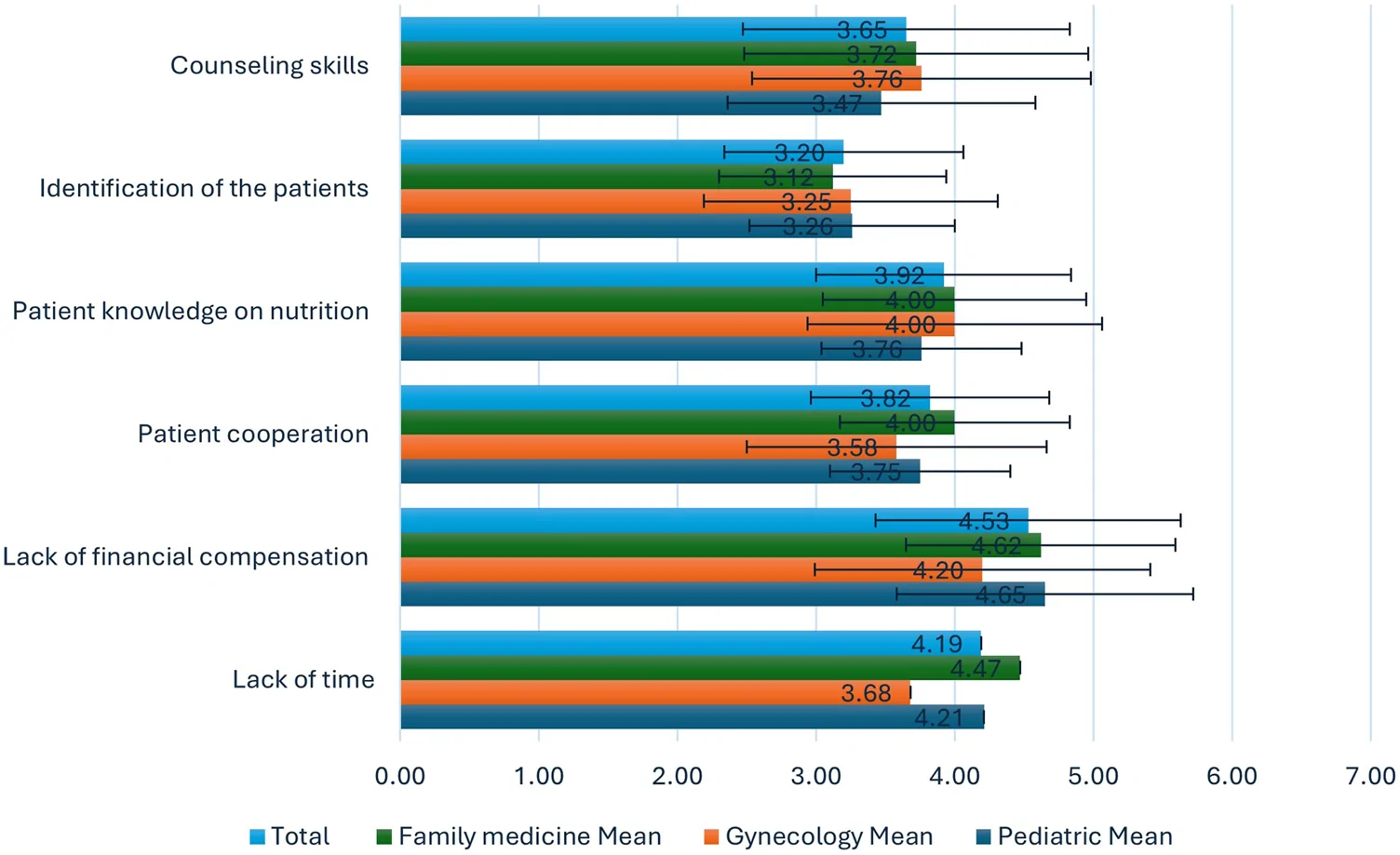
Barriers to the provision of nutritional services across departments of primary healthcare facilities (Republic of Srpska, Bosnia and Herzegovina, 2023).

### Qualitative findings

#### Assessment of the current scope and quality of nutrition services in PHCs

The qualitative analysis of the focus groups conducted with personnel from the Family Medicine, Pediatric, and Gynecology departments of the PHCs indicated differences in the current scope and practice of nutritional service provision.

In the Family Medicine departments, nutrition services were provided on an *ad hoc* basis in the form of brief nutritional advice, anthropometric measurements, and the determination of basic laboratory parameters without a clearly defined set of parameters. Nutrition counseling was mainly delivered during curative examinations of older patients suffering from chronic diseases.

“This level of service concerning counselling, I don't know how good the quality of it is and how well it is done, it's basically random advice and nothing more because of the amount of work we have, we basically say you have to take care of your diet, don't eat, and that’s what we tell them, or we just give them (patients) a piece of paper, which we already have, like a diet plan for a day or two…” (Family medicine)

“I would agree with my colleagues, we do these preventive examinations and measurements and the calculation of body mass index as part of curative examinations because due to the amount of work we do not normally schedule group meetings, so for people above the age of 18 we perform measurements during regular examinations. The people that we work more frequently with are people with chronic illnesses…” (Family medicine) [SIC]

According to the focus groups held with representatives from the Pediatric departments, nutrition services were primarily provided in the form of nutrition counseling within the counseling center for infants. Because of the increased workload, and because pediatricians in smaller health centers also work as family medicine doctors, it was not possible to dedicate separate time exclusively to nutritional services for children.

“We assess the nutritional status as part of our everyday work, we have a separate part, i.e., the counseling center where we examine children for vaccines, healthy children and during the examination we perform these services, counseling on nutrition, breastfeeding, introduction of non-dairy nutrition, what older children can eat, what they cannot, what is healthy, what is not healthy, but we really do not have a separate space and time to devote to nutrition alone. It’s not that we lack the desire for everything I've mentioned, but we don't have enough staff.” (Pediatric department) [SIC]

Insights gained through the focus groups with representatives of the Gynecology department indicated that gynecological practice at the PHC level has little contact with nutrition services. Body weight, height, body mass index, and blood pressure were measured during regular check-ups of pregnant women, and brief dietary advice was occasionally given for specific health conditions (e.g., fungal infections or obesity affecting fertility).

“What I can tell you regarding nutrition in primary healthcare in the field of gynaecology is that we do not have much to do with nutrition in terms of providing nutrition counselling in everyday practice, except that I, for example, give instruction on nutrition in cases of gynaecological infections, specifically fungal infections and the consequences that obesity can lead to, which could affect fertility in patients and in this regard we make certain corrections to the diet, not specifically us at the primary level, but we make referrals to nutritionists at the regional institute level, etc.” (Gynecology department) [SIC]

“In our everyday work we deal not only with pregnant women, but also with adolescents and children with obesity, the limit for the first menstrual cycle has moved, and it has moved because of obesity, so we now have girls coming in with their menstrual cycle established already at the age of 10 and all these things do pose real problems … ” (Gynecology department) [SIC]

#### Identified barriers to the provision of nutrition services

Similar thematic units, that is, obstacles and difficulties in providing nutrition services, were identified in the focus groups held with representatives of the Family Medicine, Pediatric, and Gynecology teams from the selected primary healthcare centers. The most frequently reported barriers were related to a shortage of medical doctors and the consequent lack of time spent with patients, in addition to the absence of guidelines with clear dietary recommendations for particular population groups and specific health conditions. Family Medicine and Pediatric personnel also stated that the existing information system does not support the planning and monitoring of preventive activities, while gynecologists noted insufficient diagnostic equipment and limited biochemical laboratory testing for women at the PHC level. Detailed thematic units across the different departments are presented in Supplementary File 1.

#### Proposals for improving nutrition services at primary healthcare centers

Representatives from the Family Medicine, Pediatric, and Gynecology departments made several proposals in the focus groups regarding the possibility of improving the quality of nutrition services at the PHC level. These focused in particular on the reorganization of nutritional and preventive services at the PHC level through multidisciplinary teams and counseling centers, with dedicated time for the provision of nutritional services and counseling to the registered population. All PHC departments stressed the importance of developing and promoting new dietary standards and guidelines, both for the general population and for specific medical conditions. In order to overcome barriers related to an insufficient number of doctors and the current workload, all departments emphasized the importance of reorganizing service provision, especially in Family Medicine. Detailed thematic units are presented in Supplementary File 2.

#### Suggestions for improving cooperation with the local community regarding the implementation of preventive activities

In the focus groups with representatives of the Family Medicine, Pediatric, and Gynecology departments, all participants from the selected PHCs underlined the importance of cooperating with the local community to conduct preventive activities and pointed to opportunities to strengthen this cooperation. Specific proposals included the proactive implementation of preventive activities in the community in accordance with the needs of the registered population (Family Medicine), the promotion of proper eating habits through social media with a focus on children and adolescents (Family Medicine), the provision of more educational materials for dissemination (Pediatric department), and the development of additional laboratory diagnostics at the PHC level (Gynecology department). Detailed thematic units with quotes are presented in Supplementary File 3.

## Discussion

Although nutrition is widely recognized as a fundamental determinant of health, it remains insufficiently prioritized within primary healthcare (PHC) systems. Despite evidence demonstrating that investing in nutrition services reduces morbidity and mortality from non-communicable diseases (NCDs), these services continue to receive limited financial support and remain below the levels required to achieve global nutrition targets [7, 9, 10].

In the Republic of Srpska, nutrition-related activities are implemented through various policies and programs [25–28]; however, this study highlights important gaps in their practical implementation at the PHC level. Consistent with previous research [17, 29, 30], our findings indicate that healthcare professionals recognize the importance of nutrition and express a clear interest in providing these services. Nevertheless, multiple systemic barriers limit their effective delivery. The most prominent challenges identified include insufficient funding, lack of time due to high workload, limited availability of guidelines, and inadequate training of healthcare professionals. These findings align with international evidence that consistently identifies time constraints, lack of knowledge, and insufficient confidence as key barriers to providing nutrition counseling in primary care [13, 14, 17, 30–32].

Although the majority of PHCs reported providing basic nutrition services, nearly one-fifth did not offer any form of nutritional monitoring or assessment. Furthermore, services were predominantly focused on counseling related to nutrition and physical activity, while services such as counseling for women of reproductive age and breastfeeding counseling were provided less frequently. This imbalance suggests that nutrition services are still largely reactive rather than comprehensive and continuous. The organization of services also varied considerably across specialist teams. Family Medicine teams played a dominant role, while Pediatric and especially Gynecology teams were less involved. In addition, coverage for women of reproductive age remained limited, despite their significant contribution to the burden of diet-related diseases [33].

A key concern identified in this study relates to inconsistencies in the application of standards and assessment methods. The limited use of dietary assessment tools and the stressed need for nutritional guidelines for specific population groups and medical disorders suggest gaps in knowledge and the absence of standardized protocols. Similar issues have been reported in other settings, emphasizing the need for clearer guidelines and structured training programs [34–36].

Financial constraints further limit service delivery. Nutrition services account for a very small proportion of total PHC funding, while preventive care in general remains underfunded compared to curative services [37, 38]. Evidence suggests that increased investment in preventive nutrition interventions could yield substantial long-term health and economic benefits [37]. This study also highlights the need for improved organization of services, including a clearer definition of roles within multidisciplinary teams, better integration of preventive activities, and the use of both individual and group counseling approaches. While individual counseling is considered more effective, group and digital approaches may improve coverage in resource-limited settings [39]. Importantly, findings from focus groups confirm that healthcare professionals face significant operational challenges, including staff shortages, a lack of tools and guidelines, and inadequate information systems. These limitations hinder the effective planning, monitoring, and evaluation of nutrition services.

This study provides valuable insight into current practices and identifies key opportunities for improving nutrition services in PHCs in the Republic of Srpska. However, several limitations should be acknowledged. First, the questionnaire relied predominantly on dichotomous response options, which constrained the analysis to descriptive rather than inferential statistics. The qualitative component used focus group discussions rather than individual interviews, a choice driven by time constraints, which allowed exploration of shared experiences across PHC. However, group dynamics may have limited the depth of individual responses compared with one-on-one interviews. Although the sample represents the entire population of public PHCs in the Republic of Srpska, supporting representativeness for the public sector, nutritional services in the private sector (particularly pediatric and gynecological practices) were not captured, which should be considered when interpreting population-level provision. The findings may also have limited generalizability to health systems with different funding models, staffing structures, or insurance arrangements, given the single-context design. Finally, self-reported responses may carry variability in accuracy, and future research should combine this approach with observational or record-based data for a more detailed assessment of specific practices.

### Conclusion

Overall, the results suggest that strengthening nutrition services in PHC requires a comprehensive approach, including capacity building among healthcare professionals, the development and implementation of standardized guidelines, improved financing mechanisms, and enhanced monitoring systems tailored to the specific profile of each department rather than applied uniformly across primary healthcare centers. The need for coordinated, system-level strengthening rather than isolated measures is consistent with recent international evidence on improving the quality of PHC services [8] and on integrating nutrition-specific interventions into routine PHC practice [18]. Greater collaboration with local communities and public health institutions is also essential in order to improve service coverage and effectiveness. Future strategies should therefore prioritize integrating multidisciplinary approaches and enhancing the use of health information systems so as to promote sustainable improvements in population nutrition and reduce the burden of NCDs.
