# Supplementary material for: Nutritional services in primary healthcare in the Republic of Srpska, Bosnia and Herzegovina: strengthening prevention through multidisciplinary approaches
Source: Int J Public Health. 2026 Oct 2;71:1609839. doi: 10.3389/ijph.2026.1609839 (PMC13672246; doi:10.3389/ijph.2026.1609839)
Supplement: Supplementary file 1 [file Table1.docx]

International Journal of Public Health

**Provision of Nutritional Services in Primary Healthcare Facilities in the Republic of Srpska, Bosnia and Herzegovina: Practices, Barriers and Potential Solutions**

Supplementary File 1. Detected barriers for provision of nutritional services among PHC departments

| Family medicine | Quotes |
| --- | --- |
| - lack of time for preventive examinations due to the high burden of curative examinations and understaffing, - lack of skills and tools (guidelines, leaflets) with specific recommendations for patients on issues concerning nutrition, - difficult access specialized nutritional services in remote municipalities, - motivation and persistence of patients during the treatment process and long waiting times for scheduled examinations, - the issue of the quality of services provided by the so-called "nutritionists" without adequate formal education, - lack of organized preventive examinations supported by active work on attracting the registered population, - lack of financing of prevention services by the Health Insurance Fund of Republic of Srpska, - deficiency of the existing IT system related to the implementation of preventive services, - burnout due to workload and working conditions. | "We have a staffing problem and as far as doctors and nurses/technicians are concerned, we are shutting down teams, and patients are just redirected to existing staff and, yes, the multidisciplinary team is great, but this too comes at the expense of family medicine teams because we are again taking someone out of family medicine and in turn this then results in problems on our side. At the level of the primary health care center, we again do not have a nutritionist or a specialist in hygiene and nutrition, and this is an additional problem.”  “I'm trying to say that the amount of work and the number of examinations reduces this possibility, and there is also the lack of skills and tools that we should have and perhaps this could be improved through workshops or organizing such Zoom lectures where we would get some accurate information, then some leaflets or pamphlets that we could give to patients."  "The issue of financing - I absolutely, absolutely agree with my colleague, I think that the emphasis should be placed on this because this volunteer work is an additional burden on the already significant workload of the primary health care centers and institutions." |
| Pediatric | Quotes |
| - lack of staff (pediatricians), with special emphasis on smaller primary health care centers where pediatricians also work as family medicine doctors, - being overburdened by the number of registered children and lack of time to devote to preventive activities and work in the counseling center, - lack of guidelines and guides on nutrition and programs for chronic non-communicable diseases, as well as certain acute health conditions, - inability to adequately use records available through the existing IT system. | “The issue of obesity, we all understand it, we know how to provide certain guidelines, etc., but we face a great deficit, not only in pediatrics but also in family medicine where I work, in terms of well-designed programs for dietary regimens for certain specific diseases and for certain specific acute conditions, this especially considering the fact that we do have some knowledge of problems brought about by chronic diseases and dietary nutrition in chronic diseases.”  "It's bad, the tables (available in the IT system) are useless because they simply cannot be used for comparison or monitoring growth and development, so that segment should definitely be improved and made more useful." |
| Gynecology | Quotes |
| - lack of staff, many registered patients in relation to the number of employed gynecologists, and lack of time for greater commitment to patients, - lack of diagnostic equipment and laboratory biochemical testing of women at the primary health care level (biochemical testing is done at the secondary level), - lack of nutritionists and nutrition sub-specialists in primary health care centers, - lack of guides with clear dietary guidelines for certain population groups and specific health conditions. | "We are facing a staff shortage, specifically we have 21,000 registered patients, and only two gynecologists..”  "I don't have clinical guidelines, I use official textbooks, I assume that a protocol for monitoring specific conditions in this area should be developed. I apologize, but if it does exist, then I too would like it to be provided a copy"  "I agree with the previous interventions of my colleagues, we are greatly hampered by this problem of laboratory biochemical testing of women in the pre-conception period, as well as all subsequent groups, in the primary sector…. because we are some 100 kilometers away from the reference center (hospital)” |
