# Supplementary material for: Nutritional services in primary healthcare in the Republic of Srpska, Bosnia and Herzegovina: strengthening prevention through multidisciplinary approaches
Source: Int J Public Health. 2026 Oct 2;71:1609839. doi: 10.3389/ijph.2026.1609839 (PMC13672246; doi:10.3389/ijph.2026.1609839)
Supplement: Supplementary file 2 [file Table2.docx]

International Journal of Public Health

**Provision of Nutritional Services in Primary Healthcare Facilities in the Republic of Srpska, Bosnia and Herzegovina: Practices, Barriers and Potential Solutions**

Supplementary File 2. Proposals for improving nutritional services at PHC level among different departments

| Family medicine | Quotes |
| --- | --- |
| - forming family medicine teams and/or multidisciplinary centers at the level of PHC that would exclusively provide preventive work - organizational possibilities for family medicine teams to periodically organize preventive work (smaller primary health care centers), - introduction of activities on planning and active encouragement of the registered population to undergo preventive examinations, - updating existing guidelines and developing new tools for health professionals to provide nutrition services, - greater availability of promotional material with expert advice for the general population and target groups of patients, - re-introduction of continuous counseling for smaller groups of patients with chronic diseases, - organization of periodic meetings with nutritional specialists with the aim of exchanging experiences and standardizing work/recommendations that will be presented to primary health care centers, - re-financing of prevention services by the Health Insurance Fund of Republika Srpska. | "I am of the opinion, and someone else mentioned this as well, that we should have teams that would exclusively deal with this issue, it can also be a nurse who would deal with the problem of nutrition for risk groups."  "I think that it would be good for the Health Insurance Fund of Republika Srpska to allocate some funds again, I think that these activities would be implemented again, regardless of the amount of work... I think that for smaller primary health care centers, the only way would be to commit ourselves, for example every 15 days, to take two hours out of our work just to perform preventive examinations."  "In family medicine, we have guidelines for obesity in children and guidelines for obesity in adults, and this represents the basis, but I think that if we want to have an impact on obesity or some other eating disorder, then we should have something more detailed, this now is the starting point for our work, but we would need more than that. I hope that this is just the beginning of our cooperation and that we will achieve our goal in this way."  "Guidelines are very important and it is important that we give standard recommendations, all of us professionals, we have clinical guidelines, but they also need to be updated and this is something we agreed on recently, ...” |
| Pediatric | Quotes |
| - the pediatric team should be responsible for a smaller number of children so that they can devote more time to each child, - children under the age of 15 should remain with the competent pediatrician, - education of health care workers, but also of parents on nutrition, - give greater importance to the profession and professionals who are specialized/educated in the field of nutrition, - promote guidelines developed in the field of nutrition (standards and norms), - development of programs and guidelines for acute and chronic health conditions. | "When we discuss what needs to be done and the issue of guidelines, or whatever you want to call them, which is something we certainly lack, then I ask that attention be paid to this as well, not only to obesity, diabetes, etc., but also to the acute and chronic conditions for which we do not have guidelines."  "You have these informally trained people, I don't know how to describe them, and patients will rather turn to them than to us because we are not publicly exposed, we do not impose ourselves, those people impose themselves with some of their advice, we are not there, we cannot express ourselves because of the amount of work we have..” |
| Gynecology | Quotes |
| - procurement of necessary diagnostic and laboratory equipment in primary health care centers (e.g. scales for assessing body composition, ultrasound device for determining osteoporosis, device for determining hormones, minerals and vitamins), - uniform dietary guidelines for all population groups, - development of clinical guidelines for eating disorders, - establishment of nutrition counseling centers at the primary health care level. | “It is important that you provide a nutrition counseling center within the primary health care center, or rather, that there is a nutritionist who would provide not only women of reproductive age, but also the general population with some guidelines and adequate advice regarding nutrition…”  "A donation of laboratory equipment that would be used for diagnostics is essential, and of course, hiring nutritionists and sub-specialists that would help us. We don't have scales for assessing body composition at the primary level.” |
