# Supplementary material for: Nutritional services in primary healthcare in the Republic of Srpska, Bosnia and Herzegovina: strengthening prevention through multidisciplinary approaches
Source: Int J Public Health. 2026 Oct 2;71:1609839. doi: 10.3389/ijph.2026.1609839 (PMC13672246; doi:10.3389/ijph.2026.1609839)
Supplement: Supplementary file 3 [file Table3.docx]

International Journal of Public Health

**Provision of Nutritional Services in Primary Healthcare Facilities in the Republic of Srpska, Bosnia and Herzegovina: Practices, Barriers and Potential Solutions**

Supplementary File 3. Suggestions for improvement of implementation of preventive services in local settings by different departments

| Family medicine | Quotes |
| --- | --- |
| - establishing a uniform method of work related to the planning and implementation of promotional and preventive activities in the community, - establishing cooperation with parents' councils in schools with the aim of promoting services provided by primary health care centers, - proactive implementation of preventive activities in the community in accordance with the needs of the registered population, - strengthening active cooperation with associations of patients with various chronic diseases, - promotion of proper eating habits through social media with a focus on children and adolescents, - limiting the availability and advertising of nutrient-poor and energy-dense foods to children (near schools and preschool facilities). | "The door is open and the institutions are interested in cooperation, but we are missing a uniform method of work whereby we would all work equally, when such lectures are organized. We would then all have similar presentations and guidelines aimed at certain target groups, so in addition to children, it would also be good to establish cooperation with parents through parent councils in schools.”  “In addition to this form of promotion, which the doctor has just explained, namely the promotion of healthy habits using social media because this is how young people now live, I also read an excellent example, not from our region, but from Europe, where the local community, i.e., the city does not allow small fast-food shops and bakeries to operate near elementary schools…” |
| Pediatric | Quotes |
| - more promotional and technical materials and their dissemination, - introduction of school cafeterias, - introduction of nutrition schools at the primary health care level (similar to maternity schools at primary health care centers). | "Definitely more promotional and technical material and its dissemination, more meetings to be organized, more opportunities for exchanging experiences and sharing those promotional materials."  Nutrition schools, like schools for pregnant women, that would be really nice for the children, if parents and children could come, if there was someone to do that, that would be really nice..." |
| Gynecology | Quotes |
| - development of additional laboratory diagnostics at the primary health care center level, - financial stimulation for preventive work. | “This cooperation with the city is certainly yielding results, but we need a lot more, also in money, financial resources from the city and the Fund (Health Insurance Fund). …. Of course, we would all like to be additionally rewarded for preventive work, because it is usually volunteer work or very low-paid work during the weekends." |
